# Diethylcarbamazine activates TRP channels including TRP-2 in filaria, *Brugia malayi*

**DOI:** 10.1038/s42003-020-01128-4

**Published:** 2020-07-28

**Authors:** Saurabh Verma, Sudhanva S. Kashyap, Alan P. Robertson, Richard J. Martin

**Affiliations:** Department of Biomedical Sciences, Iowa State University, Ames, IA 50011 USA

**Keywords:** Parasitic infection, Pharmacodynamics

## Abstract

Diethylcarbamazine is an important classic drug used for prevention and treatment of lymphatic filariasis and loiasis, diseases caused by filarial nematodes. Despite many studies, its site of action has not been established. Until now, the consensus has been that diethylcarbamazine works by activating host immune systems, not by a direct action on the parasites. Here we show that low concentrations of diethylcarbamazine have direct and rapid (<30 s) temporary spastic paralyzing effects on the parasites that lasts around 4 h, which is produced by diethylcarbamazine opening TRP channels in muscle of *Brugia malayi* involving TRP-2 (TRPC-like channel subunits). GON-2 and CED-11, TRPM-like channel subunits, also contributed to diethylcarbamazine responses. Opening of these TRP channels produces contraction and subsequent activation of calcium-dependent SLO-1K channels. Recovery from the temporary paralysis is consistent with inactivation of TRP channels. Our observations elucidate mechanisms for the rapid onset and short-lasting therapeutic actions of diethylcarbamazine.

## Abstract (web summary)

Verma et al. describe a new mechanism for diethylcarbamazine action, a drug classically used to treat lymphatic filariasis and loiasis. They propose that it induces rapid temporary spastic paralysis of parasites by opening TRP channels, including TRP-2 in muscle of *Brugia malayi*. Inactivation of TRP channels explain its short-lasting therapeutic activity.

## Introduction

The neglected tropical diseases include infections like loiasis and lymphatic filariasis caused by filarial nematode parasites like *Loa loa, Brugia* spp., and *Wucheria bancrofti*. The diseases are transmitted by biting insects that feed on infected hosts’ blood, picking up L1 stage microfilaria, which mature in the insect to the L3 stage, before being passed on to a subsequent uninfected host. The transmitted microfilariae mature in the new host to adults where the clinical signs produced depend on the final location of the parasites. Loiasis, or African eye worm, is caused by the filarial worm *L. loa* that threatens more than 29 million people causing itchy swellings of the body known as Calabar swellings. A group of filarial adults like *Brugia malayi* that locate in the host lymphatic system produces lymphatic filariasis. Here, they block drainage of the lymphatics inducing gross swelling of the limbs, itching, and skin infections that produce the clinical condition, elephantiasis. The unpleasant symptoms inhibit productive work of the individual and often cause social rejection. There are an estimated 67.88 million people infected with lymphatic filariasis^1^, which threatens 886 million people in 52 countries. There are no effective vaccines, so Mass Drug Administration (MDA) to control and prevent infection is the only practical option. Diethylcarbamazine (referred to subsequently as DEC), is a valuable and classic drug^2^ used for the treatment of loiasis and lymphatic filariasis^3,4^; it has a very fast therapeutic onset; it removes microfilariae from the blood, but the therapeutic benefits are temporary because the microfilariae return in the blood after only a few hours^5^.

Until now, DEC was not understood to have direct effects on the parasites, but taken to act by stimulating host immune systems^6–8^. We have discovered that low concentrations of DEC have a direct effect on *B. malayi* parasites, opening Transient Receptor Potential (TRP) channels, which in turn activate calcium-dependent SLOw poke potassium channels (SLO-1) in their somatic muscle cells, and produce a temporary spastic paralysis. The identification of this site of action of DEC in filaria can explain the rapid onset of action in vivo following administration and its transient therapeutic effect that can be followed by recovery of infection levels after treatment. An entry of calcium and activation of SLO-1 channels by DEC also predicts a synergistic interaction between DEC and emodepside that is under development for treatment of filaria. The new knowledge of the mode of action of DEC will support its continued use and needed combination therapies.

## Results

### DEC effects on microfilariae and adult motility

**Fig. 1:**
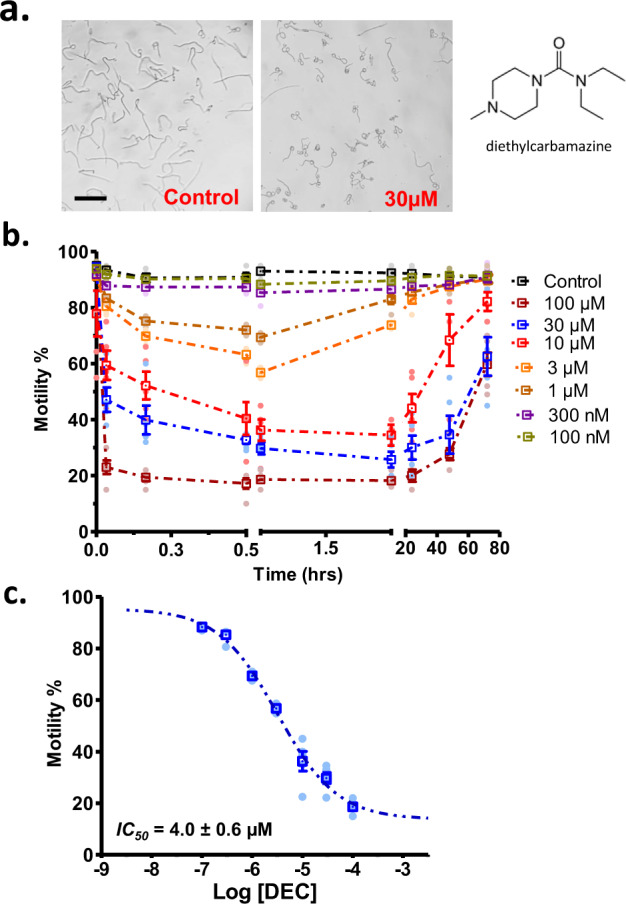
DEC concentration effects on microfilaria of *Brugia malayi*. **a** Photomicrographs of effects of control (no drug added) and 30 µM DEC. Note the difference in appearance between the control and DEC treatment which resulted in looping, coiling and reduction in motility. Worms were considered non-motile for analysis if the worms were fully contracted or demonstrated tail or head coiling. (Scale bar = 200 µm). **b** The time-dependent and concentration-dependent effects of DEC. Note that DEC produces a concentration-dependent inhibition of motility that is present at the first recording of 2 min and that the inhibition of motility at the higher concentrations declines (the microfilaria recovers) after 0.5 h with 1 and 3 µM. Motility also recovers even with higher DEC concentrations but takes longer. We used three separate deliveries from FR3; placed 40 microfilaria in each well and followed over time the effects in 5 wells for each concentration. We had 200 microfilariae for each concentration. For some concentrations, the standard error (SE) plotted are smaller than represented points on the graph. **c** The DEC concentration-dependent effect on the % motile of microfilaria at 1 h. The IC_50_ was 4.0 ± 0.6 µM (three separate deliveries from FR3 and a total of *n* = 200 microfilariae for each concentration with seven concentrations of DEC and a zero control); for some concentrations the SE plotted are smaller than represented points on the graph.

**Fig. 2:**
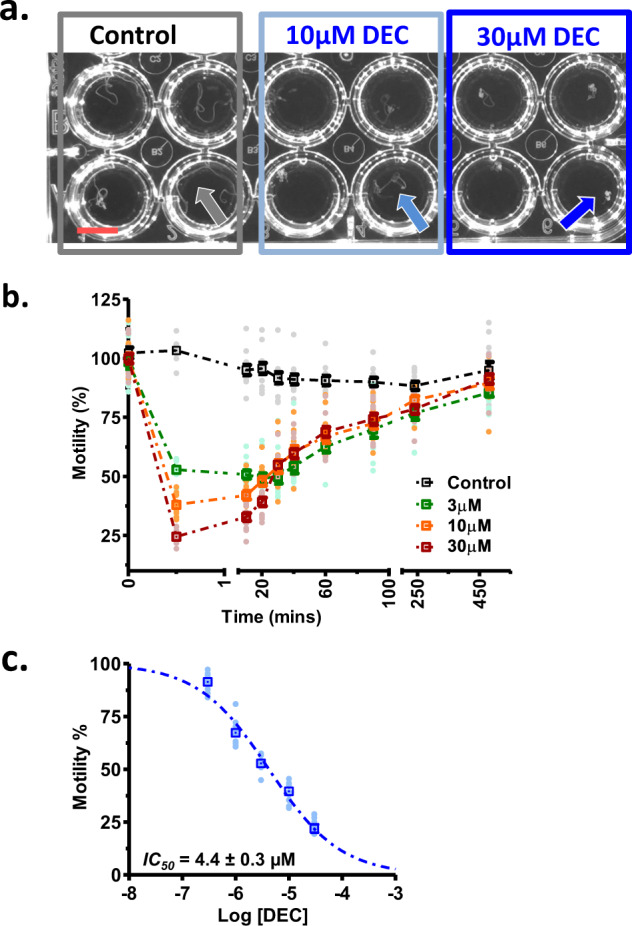
The concentration-dependent motility inhibitory effect of DEC on adult female *B. malayi* in 24-well plates and their motility recorded with the Worminator system. **a** Control, 10 and 30 µM DEC effects on adult *B. malayi* at 30 s. In the control, the worms move normally (gray arrow): in 10 µM they are somewhat curled (pale blue arrow); in 30 µM (darker blue arrow) they are curled into a ball (scale bar = 0.5 cm). **b** Normalized motility plots over time from the Worminator following addition of different concentrations of DEC. Note the concentration-dependent differences and the recovery over time (*n* = 12 worms per concentration, from four deliveries of FR3 worms; total worms 60 adult females). **c** The DEC concentration-dependent effect on the inhibition of motility at 30 s from the worms shown in **b**. The IC_50_ was 4.4 ± 0.3 µM (*n* = 12 worms per concentration, 5 concentrations from 4 deliveries of FR3 worms; total worms 60 adult females).

We tested the effects of addition of different concentrations of DEC on the motility of microfilariae of *Brugia malayi* (Fig. 1a, b). DEC produced rapid (<2 min) coiling, loops, and inhibition of motility that was concentration-dependent. These effects were followed by a gradual recovery that started after 30 min at 1 µM and 24 h at 100 µM^9^. The % motile IC_50_ at 30 min was 4.0 ± 0.6 µM (*n* = 200/concentration) (Fig. 1c). We tested the effects DEC on the motility of adult female *B. malayi* (Fig. 2a, b), and found that DEC produced a fast (<30 s) contraction and spastic paralysis that was concentration-dependent and that lasted ~3 h before a gradual recovery over the next 5 h. The IC_50_ for the % motility inhibition at 30 s was 4.4 ± 0.3 µM (*n* = 12) (Fig. 2c).

### DEC effects on muscle currents

**Fig. 3:**
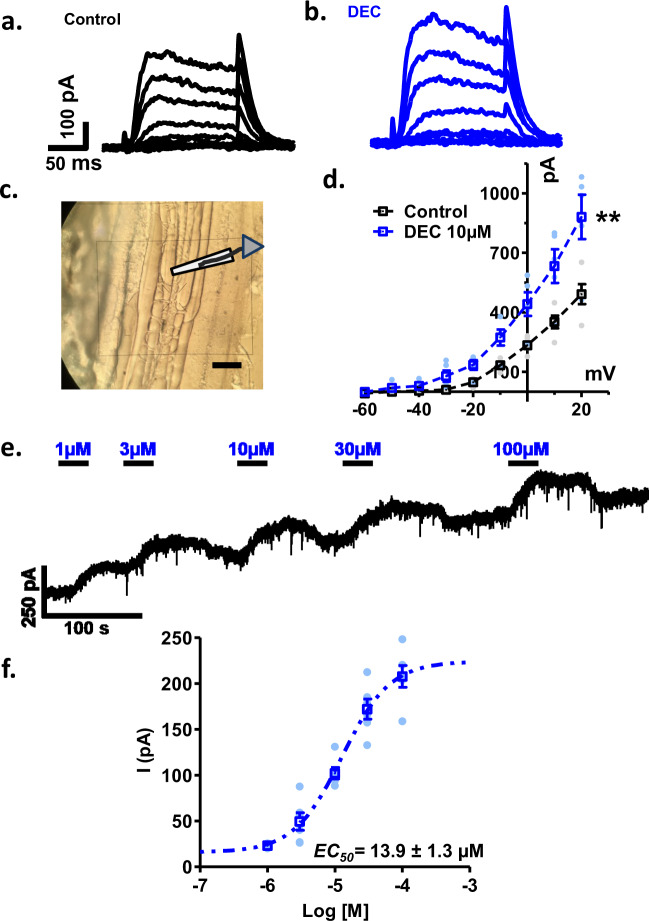
Effect of DEC on voltage-activated and standing outward currents of *B. malayi* somatic muscle under whole-cell voltage-clamp. **a** Control and **b** DEC voltage-activated currents, holding potential −40 mV and effects of step potentials incremented by 10 mV from −60 mV to +20 mV, black are the control currents while blue is the currents in presence of 10 µM DEC. **c** Photomicrograph of a *B. malayi* muscle flap under Nomarski optics and the position of a patch-pipette included (scale bar = 10 mM). **d** I–V plot of the voltage-activated currents. Each current is the mean of five observations from five worms. Black line represents control while blue line represents observations in presence of 10 µM DEC. Note the increase in size of the current with DEC. The current at +20 mV step increased from 492 ± 50 pA to 881 ± 112 pA in presence of DEC (paired *t*-test, *p* = 0.007; 95% confidence interval was −503.1 to −74.13; *n* = 5 muscles from five worms). **e** The holding currents at a holding potential of −40 mV during 25-s applications of increasing concentrations of DEC. Note that the response to DEC is relatively fast, within 10 s following application. The effect of each concentration of DEC is not fully reversed on washing. **f** DEC-concentration peak outward current response plot. EC_50_ 13.9 ± 1.3 µM (*n* = 6 from five worms).

We examined the effects of 10 µM DEC on the electrophysiology of the body wall muscle cells under whole-cell patch-clamp in 6 separate muscle cells from five different B*. malayi* female worms. We found in each of the preparations that DEC increased a voltage-sensitive outward current (Fig. 3a–d), similar to what we had previously described in *Ascaris suum*, to include a calcium-dependent SLO-1 K current^10^. In addition to the effects on the voltage-sensitive outward current, the main effect of DEC was to produce a concentration-dependent increase in the standing outward current (Fig. 3e), that had an EC_50_ of 13.9 ± 1.3 µM (*n* = 6) (Fig. 3f). This outward potassium current was inhibited by the addition of iberiotoxin (30 µM), a selective inhibitor of calcium-dependent BK (SLO-1 K) channels (Supplementary Fig. 1).

**Fig. 4:**
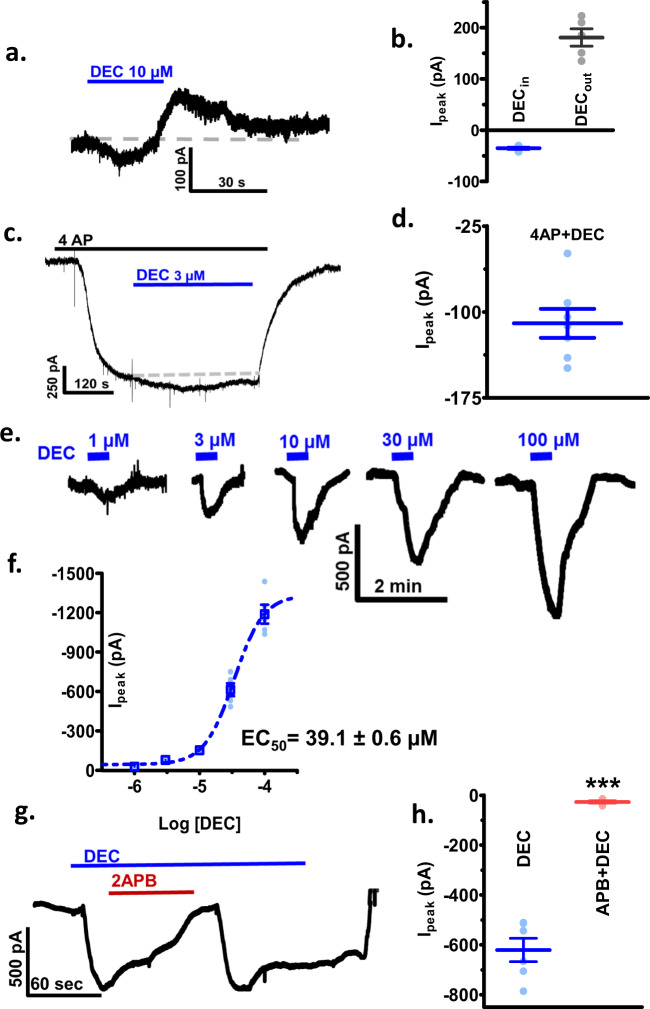
DEC induced inward currents at −40 mV revealed by blocking SLO-1 K currents with 5 mM 4AP. **a** Representative DEC biphasic holding currents, in the absence of 4AP, following the application of 10 µM DEC at −40 mV. Note that an inward (downward) current precedes the outward current. **b** Box-plot representing mean ± SE of the initial inward peak current (−35.2 ± 2 pA) and the subsequent peak outward current (180.8 ± 16.8 pA) following 10 µM DEC (*n* = 5 from five worms). **c** Application of 5 mM 4AP produces a steady inward current due to the blocking of the steady outward potassium current. The application of 3 µM DEC during the plateau of the 4AP effect produces a steady inward current. **d** Box-plot of the mean 3 µM DEC inward current during the plateau of the 4AP effect, −109.9 ± 12.6 pA (*n* = 7 muscles from five worms). **e** DEC concentration-dependent inward current-responses in the presence of 5 mM 4AP at a holding potential of −40 mV. **f** Plot of the DEC concentration-dependent inward current-responses in the presence of 5 mM 4AP, −40mV, EC_50_ 39.1 ± 0.6 µM (*n* = 5 muscles from five worms; for some concentrations the SE plotted are smaller than represented points on the graph). **g** Application of 2APB (30 µM) as an inhibitor of TRP channels inhibits the inward DEC (30 µM) current seen in the presence of 5 mM 4AP. **h** Box-plot of the 30 µM DEC induced inward currents before (−620.9 ± 47 pA) and in the presence of 30 µM 2APB (−26.9 ± 4.7 pA) as an inhibitor of TRP channels (paired *t*-test, *p* = 0.0001; 95% confidence interval was −720 to −470; *n* = 7 from six worms).

Upon closer inspection of the standing currents caused by DEC, we observed in many recordings, an inward current that preceded the outward SLO-1 K current (Fig. 4a, b). These inward currents suggest that DEC activates SLO-1 by activating an inward current that carries calcium through the plasma membrane. This entry of calcium through the membrane increases SLO-1 K currents and can also explain the contraction and spastic paralysis produced by DEC.

### Evidence of TRP channels

To study these inward currents further, we blocked the outward SLO-1 K currents by using 4-aminopyridine (4AP), a potassium channel antagonist^10^. The application of 5 mM 4AP inhibited the potassium currents and produced a significant inward current, which reached a steady level after 1.5 min. Following the addition of the 4AP, when a steady current was reached, the application of DEC produced a steady inward current (Fig. 4c, d). In the presence of 4AP, we found that DEC produced a concentration-dependent reversible inward current with an EC_50_ of 39.1 ± 0.6 µM (*n* = 5) (Fig. 4e, f). This inward current was reversibly inhibited by 10 µM 2APB (Fig. 4g, h), and 10 µM SKF96365, (Supplementary Fig. 2), both non-selective TRP channel blockers^11^, suggesting that the inward current produced by DEC is due to opening of TRP channels.

TRP channels are a group of non-selective tetrameric cation channels permeable to cations including sodium, potassium, and calcium. We were able to inhibit the SLO-1 K channel currents of *B. malayi* muscle induced by DEC by perfusing the preparations with calcium-free bath solution (Supplementary Fig. 3). Under these conditions, application of 30 µM DEC produces only an inward current likely carried by sodium and potassium through open non-selective TRP channels but cannot increase intracellular calcium to activate the SLO-1K channels. However, returning the calcium to the perfusing bath solution allowed activation of the SLO-1K channels with the entry of calcium through the TRP channels (Supplementary Fig. 3).

As a further test for the presence of TRP channels in *B. malayi* muscle cells, we tested capsaicin that activates TRPV1 channels in vertebrates^12^. We found that the effect of 30 µM capsaicin in the presence of 4AP was also able to induce an inward current like 30 µM DEC (Supplementary Fig. 4). The effect of co-application of 30 µM DEC and 30 µM capsaicin was to produce inward currents that were additive rather than mutually exclusive, suggesting that the two compounds do not act on the same receptor but on different types of TRP channels.

### Expression of TRP channels in adults and microfilaria

**Fig. 5:**
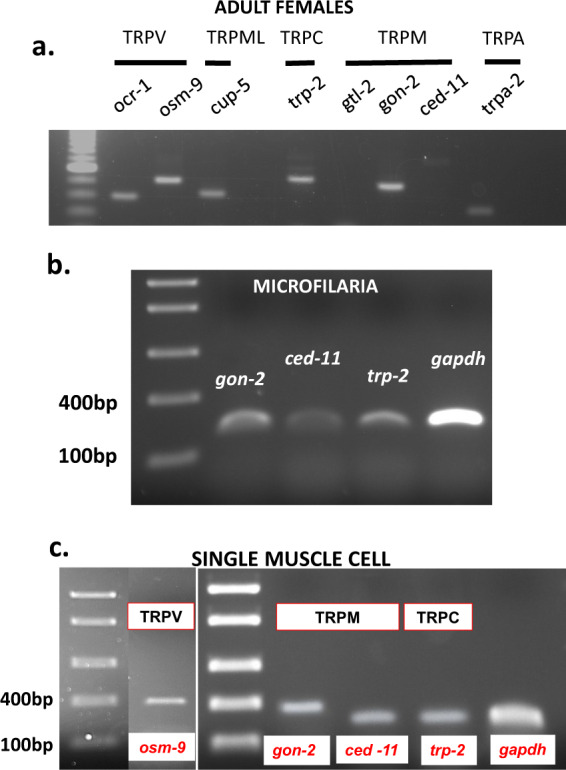
Identification of TRP channel subtypes in the *B. malayi* whole adult female, microfilaria, and single muscle cells. **a** RT-PCR showing the presence of TRPV (*ocr-1* and *osm-9*), TRPML (*cup-5*), TRPC *(trp-2*), TRPM *(gon-*2 *and ced-*11), and TRPA (*trpa-*2) in whole adult females (*n* = 5). **b** Expression of *gon-2, ced-11,* and *trp-2* in microfilaria. RT-PCR was performed on five different batches of microfilaria and shown here is a representative figure (*n* = 10^6^ microfilaria). **c** Single muscle cell PCR demonstrating the presence of different TRP channel subtypes expressed in a single muscle cell. Representative gel picture shows the presence of TRPV (*osm-9*), TRPM (*gon-2, ced-*11), and TRPC (*trp-*2) subtypes. *gapdh* was the internal control for each cell (*n* = 6 from six worms).

We used primers for PCR, based on the genome of *B. malayi*, to detect expression of TRP channel transcripts in whole adult female worms (Fig. 5a), and found *ocr-1, osm-9, cup-5, trp-2*, *gon-2, ced-11*, and *trpa-2* transcripts. We observed expression of *trp-2*, *gon-2*, and *ced-11* in microfilariae but did not detect expression of other TRP channel transcripts (Fig. 5b).

In order to identify TRP channels expressed in single muscle cells of adults, we performed RT-PCR on cytoplasm collected from single muscle cells with a patch pipette^13,14^. As shown in Fig. 5c: *osm-9, gon-2, ced-11,* and *trp-2* were expressed in muscle cells. OSM-9 are TRPV-like channel subunits and may explain responses of muscle cells to capsaicin (Supplementary Fig. 4); TRP-2s are TRPC-like channel subunits; and GON-2 and CED-11 are TRPM-like channel subunits.

### TRP channel subunits required for DEC responses

**Fig. 6:**
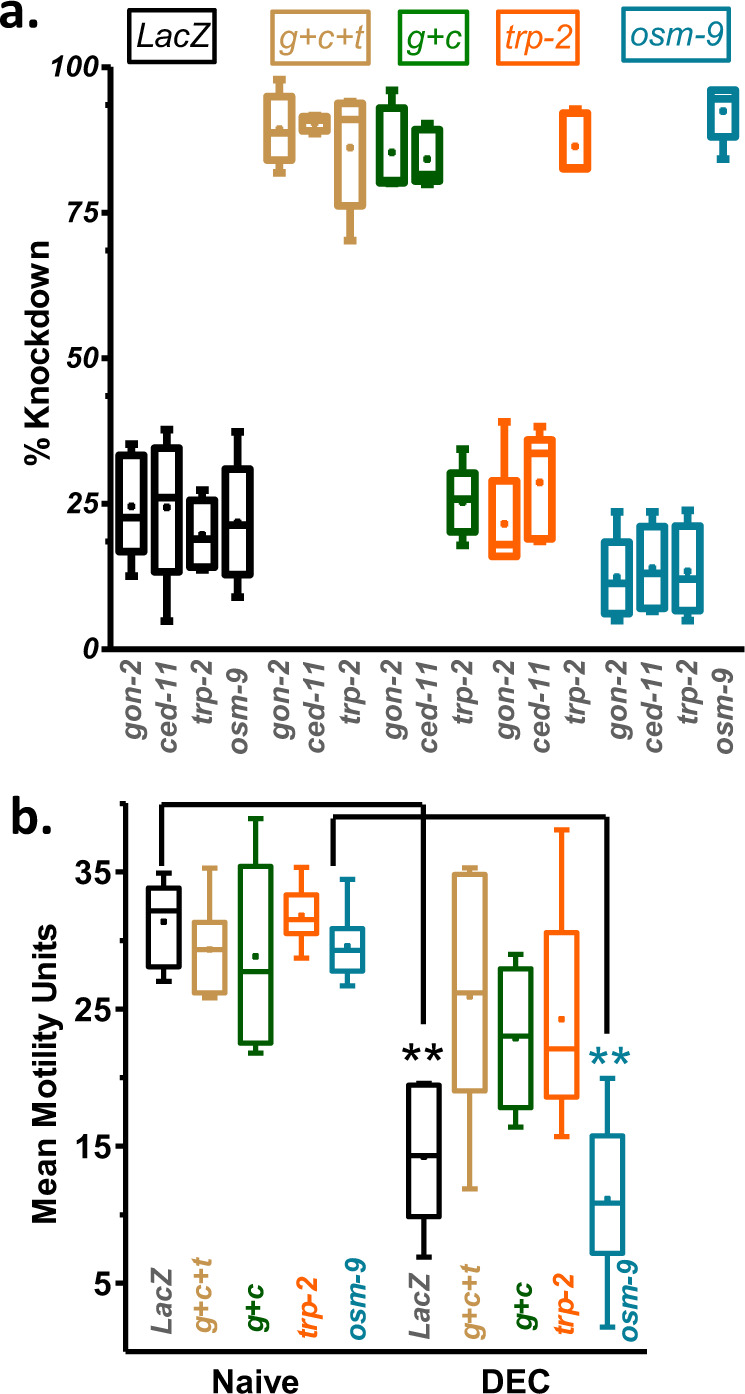
Loss of a TRP-2, a GON-2, and CED-11 but not OSM-9 channels causes resistance to DEC. **a** Box-whisker plot for quantitative PCR demonstrates significant reduction in transcript level of individual subtypes in dsRNA-treated *B. malayi*. Box-whisker plot demonstrating quantitative PCR results of non-significant reduction in transcript levels of *gon-2, ced-11*, and *trp-2* compared to control transcript levels in LacZ dsRNA-treated worms, *gon-2* by 24.6 ± 4.0%, *ced-11* by 24.4 ± 5.4%, trp-2 by 19.6 ± 2.6%, and *osm-*9 by 21.7 ± 4.6 and significant reduction in *gon-2* + *ced-11* + *trp-2* dsRNA-treated worms, *gon-2* by 89.3 ± 2.7%, *ced-11* by 90.4 ± 0.6% and *trp-2* by 86.6 ± 4.5%, respectively. *gon-2* + *ced-11* (TRPM-like) dsRNA treatment lead to specific reduction in *gon-2* by 85.3 ± 3.2% and *ced-11* by 84.2 ± 2.1% transcripts only. *trp-2* (TRPC-like) dsRNA treatment specifically knocked down *trp-2* transcript by 86.4 ± 2.3%. While *osm-9* (TRPV-like) dsRNA treatment specifically knocked down *osm-9* transcript by 92.4 ± 2.0% (*n* = 5 from three batches). **b** Box-whisker plot showing selective loss of effect of DEC on motility for *Gon-2, ced-11,* and *TRP-1* dsRNA-treated worms. Control worms were significantly paralyzed after DEC treatment, the mean motility decreased from 31.4 ± 1.3 to 14.2 ± 2, whereas dsRNA-treated worms were not affected (*g* + *c* + *t: gon-2* + *ced-11* + *trp-2*, 29.4 ± 1.4 to 23 ± 3 MMU); (*g* + *c: gon-2* + *ced-11*, 29 ± 2.7 to 23 ± 2 MMU), and (*trp-2:* 31.8 ± 0.8 to 24.2 ± 3.2 MMU). Two-way ANOVA with Bonferroni post-hoc test; *p* = 0.001; 95% confidence interval for *LacZ* was −30 to −9; 95% confidence interval for *osm-9* was −30 to −10; *n* = 6 from three batches. Knockdown of *osm-9*, caused no change in the sensitivity of worms to DEC with no change in mean motility between the control worms and *osm-9* dsRNA-treated worms. Mean motility unit (MMU) is an arbitrary unit defined as the average pixel displacement over time measured in a well containing a worm using the Worminator^35^.

In order to identify which TRP channel subunits are required for the motility inhibitory response to DEC, we used dsRNA exposure for 4 days to knockdown *osm-9*, *gon-2*, *ced-11*, and *trp-2* (Fig. 6a). Knockdown of: *gon-2* + *ced-11* + *trp2, gon-2* + *ced-11*, or *trp-2* but not *osm-9* produced a significant loss of the inhibitory effect of 30 µM DEC on motility (Fig. 6b). These observations suggest that TRP channels with TRP-2, CED-11, and/or GON-2 channel subunits are activated by DEC to produce the spastic paralysis that inhibits motility of adult *B. malayi*.

**Fig. 7:**
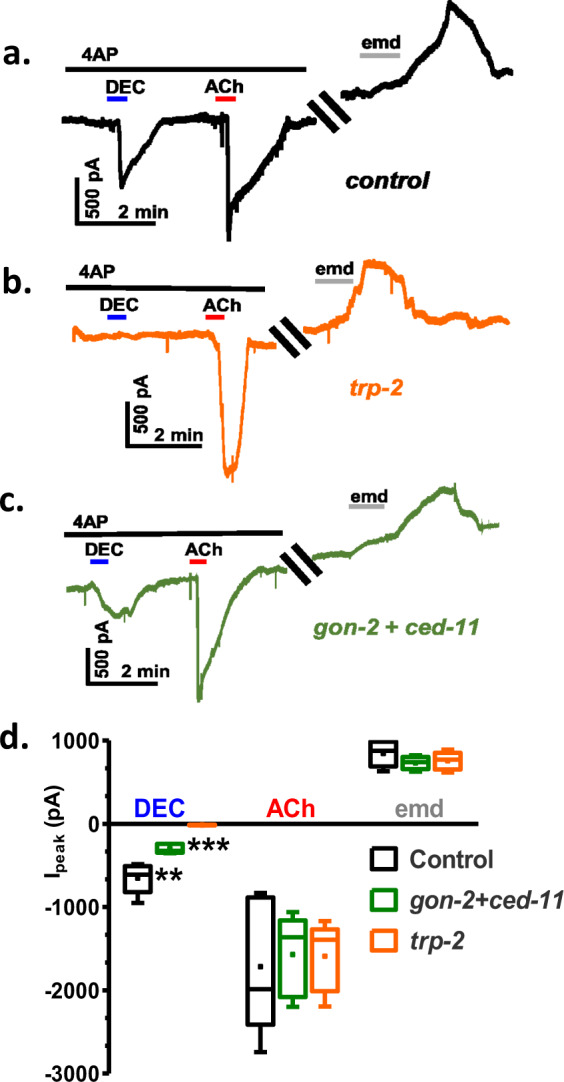
Effect of knockdown of TRP-2, and GON-2 + CED-11 TRP channels on the DEC activated inward currents with control acetylcholine (ACh), and emodepside (emd) current-responses. **a** Control worms: inward currents induced in the presence of 5 mM 4AP by 30 µM DEC (−653 ± 81 pA, *n* = 8 muscles) and acetylcholine induced (−1716 ± 36 pA, *n* = 8). In absence of 4AP, emodepside produced outward current (846 ± 69 pA, *n* = 8 muscles). **b** TRP-2 dsRNA knockdown worms: currents induced in the presence of 5 mM 4AP, 30 µM DEC (−14.5 ± 1.5 pA, *n* = 8) and ACh induced (−1590 ± 185 pA, *n* = 8) inward currents. In absence of 4AP, emodepside produced outward current (756 ± 47 pA, *n* = 8). Note the striking inhibition following TRP-2 knockdown. **c** GON-2+CED-11 dsRNA knockdown worms: currents induced in the presence of 5 mM 4AP, 30 µM DEC (−300 ± 24 pA, *n* = 8) and ACh induced (−1570 ± 217 pA, *n* = 8) inward currents. In the absence of 4AP, emodepside produced outward currents (732 ± 34.6 pA, *n* = 8). **d** Box-whisker plot demonstrating the effect of different dsRNA treatments on DEC, ACh, and emodepside induced currents. The inward DEC currents were significantly inhibited by the TRP-2 knockdown and the CED-11+GON-2 knockdown. The biggest effect was observed for the TRP-2 knockdown (one-way ANOVA with Bonferroni post-hoc test; *p* < 0.0001, 95% confidence interval for control vs. *gon-2* + *ced-11* was −571.6 to −134.2 and for control vs. *trp-2* was −857.2 to −419.9; *n* = 5 from five worms).

Because worm motility is governed by both somatic muscle and neuronal activity and *trp-2, ced-11,* and *gon-2* may have a different role in nerves and muscle, we examined the effect of dsRNA knockdown on DEC activated currents in muscle, again in the presence of 4AP. Fig. 7a shows, in the presence of 4AP, that effects of DEC on control worms are to produce inward currents by opening TRP channels, and the effects of acetylcholine by opening nicotinic receptor channels is also to produce inward currents. Following the removal of 4AP, emodepside (a SLO-1K channel agonist) produces outward currents. Fig. 7b, d shows that the effect of *trp-2* knockdown is to block the effect of DEC without reducing the acetylcholine or emodepside induced currents. Fig. 7c, d shows that the effect of *ced-11* + *gon-2* knockdown is to produce a significant reduction but not a block of the effect of DEC: the effect of *trp-2* knockdown is ~2× that of *ced-11* + *gon-2* knockdown response. The responses to acetylcholine and emodepside were not significantly affected by the knockdown. Thus, the DEC current-responses require TRP-2 subunits; GON-2, and CED-11 support the DEC response. These observations can be explained if DEC activates heteromeric TRP channels^15^ in *B. malayi* muscle with TRP-2 subunits that are essential and that includes GON-2 or CED-11 subunits as optional subunits.

### Arachidonic acid, lipoxygenases, cyclooxygenases, and PUFAs

The inhibition of lipoxygenase and/or cyclooxygenase that block the synthesis of physiologically active prostanoids in the host and parasites has been discussed as a mechanism of action of DEC^6–8^. But we now know that particular nematodes do not have cyclooxygenases or lipoxygenases: *C. elegans* and *B. malayi* lack identified cyclooxygenases or lipoxygenases in the databases (WormBase).

Nevertheless, nematodes still produce poly-unsaturated fatty acids (PUFAs) with two types of cytochrome oxidase CYP450 enzymes acting on arachidonic acid: (1) ω-hydroxylases producing 20-hydroxyeicosatrienoic acids (HETEs); and (2) epoxygenases producing epoxyeicosatrienoic acids (EETs)^16^. The PUFAs produced by these CYP450 enzymes can explain the ability of *B. malayi* to produce prostacyclin, PGE_2_ and PGD_2_ from arachidonic acid in the absence of cyclooxygenases and lipoxygenases^17,18^. In *B. malayi*, the homolog of the mammalian ω-hydroxylase is BM42071, and the homolog of the mammalian epoxygenases that are inhibited by miconazole is BM38240^19^. Thus, it was possible that DEC acted by inhibiting CYP450 enzymes.

**Fig. 8:**
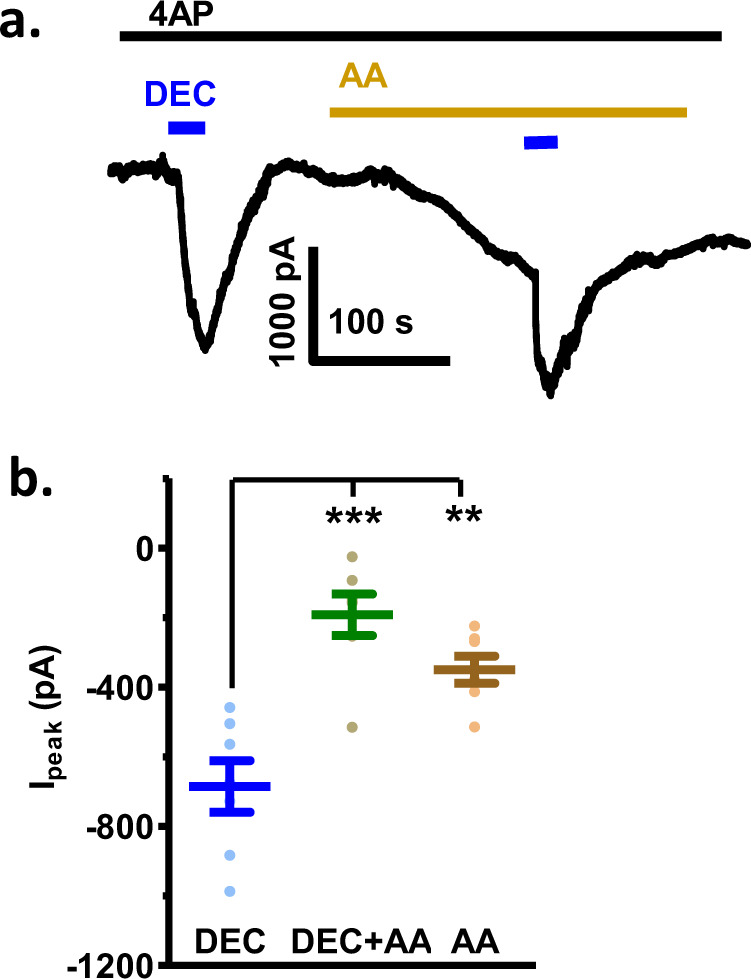
Arachidonic acid slowly increases the inward current and reduces the peak response of 30 µM DEC in the presence of 5 mM 4AP to block SLO-1 K currents. **a** Representative inward currents showing that the application of 30 µM arachidonic acid produces a slowly increasing inward current that reduces the peak response to 30 µM DEC (5 mM 4AP present to block SLO-1 K). **b** Whisker-plot of peak inward currents induced by 30 µM DEC (DEC: −685.5 ± 74 pA), 30 µM arachidonic acid (AA: −350 ± 39 pA), and 30 µM DEC in the presence of 30 µM arachidonic acid (DEC + AA: −192 ± 60 pA). The peak DEC + AA current response, and the AA current response are significantly less than the peak DEC alone current (one-way ANOVA with Bonferroni post-hoc test; *p* < 0.0001 for DEC vs. DEC + AA, 95% confidence interval was −700 to −300; and *p* = 0.008 for DEC vs. AA, 95% confidence interval was −600 to −100).

We tested the effect of arachidonic acid and found that its effect was similar to DEC (Fig. 8a, b), but the onset was much slower than DEC, taking several minutes to plateau. This suggests metabolism of arachidonic acid, to an active metabolite was required to produce the effect on the TRP channels. The peak amplitude of the current response to DEC was also reduced in the presence of arachidonic acid that may be explained by both the arachidonic acid metabolite and DEC acting as agonists on the same TRP channels.

### Meclofenamic acid, indomethacin, and miconazole

Next, we tested the effect of 30 µM meclofenamic acid, an inhibitor of mammalian 5-lipoxygenase and cyclooxygenase and found Supplementary Fig. 5a, b, no effect on the DEC induced currents. We also tested the effect of 30 µM indomethacin as a cyclooxygenase inhibitor and found that it also produced no significant effect on the amplitude of the response to 30 µM DEC (Supplementary Fig. 5c, d).

**Fig. 9:**
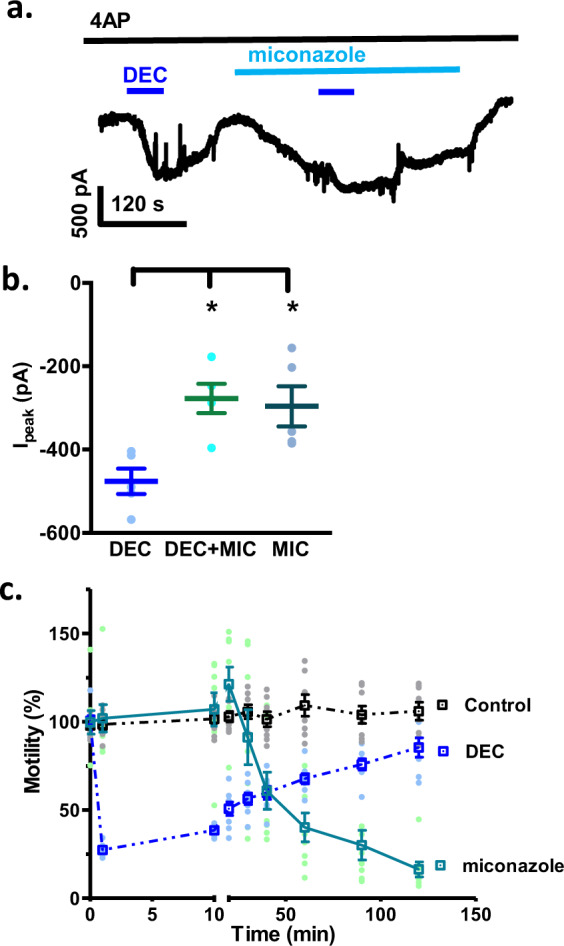
Effects of miconazole on DEC induced inward currents and worm motility. **a** Representative inward currents following the control application of 30 µM DEC followed by the application of 10 µM miconazole and again 30 µM DEC in presence of miconazole. 5 mM 4AP present throughout. **b** Whisker-plot of the net inward currents induced by 30 µM DEC (−476 ± 30 pA), 10 µM miconazole (−277 ± 35 pA) and 30 µM DEC + 10 µM miconazole (−296 ± 48 pA). One-way ANOVA with Bonferroni post-hoc test; *p* = 0.006; 95% confidence interval for DEC vs. DEC + miconazole was −400 to −50 pA and for DEC vs. miconazole was −300 to −30 pA; *n* = 5 from five worms. The DEC-induced current is similar in amplitude to DEC + miconazole induced inward currents. **c** Normalized motility plots over time from the Worminator following addition of 30 µM DEC and 10 µM miconazole. Note that both of them produced different responses: DEC produced a spastic paralysis initially followed by recovery; miconazole produced a delayed flaccid paralysis with no recovery (*n* = 8 worms from two different batches of worms).

With no effect of meclofenamic acid and indomethacin on the amplitude of the peak DEC responses, we tested 10 µM miconazole as an inhibitor of epoxygenase CYP450 enzymes. We found that, like arachidonic acid, miconazole produced a slow inward current and reduced the amplitude of the response to DEC (Fig. 9a, b), suggesting that the inhibition of epoxygenases may divert the metabolism of arachidonic acid through hydroxylases^20^ to metabolites that also can activate the TRP-2 channels. The current-responses to both arachidonic acid and miconazole were slow, taking several minutes to plateau, in contrast to the faster action of DEC. The slower time course of arachidonic acid and miconazole can be explained if accumulation of metabolites arachidonic acid are required for activation of *Brugia* TRP channels and if DEC acts directly on these TRP channels.

As a further test of the hypothesis that miconazole and DEC do not have an identical mode of action (i.e., both inhibit specific CYPs), we compared the effects of the two drugs on motility (Fig. 9c). We found that the effect of miconazole was slow in onset, taking 10 min for an effect, which was to produce a slowly increasing flaccid paralysis without a recovery. DEC, in contrast, produced a rapid onset spastic paralysis that was followed after a few hours by recovery. We concluded that miconazole and DEC do not have identical modes of action on *B. malayi*.

## Discussion

**Fig. 10:**
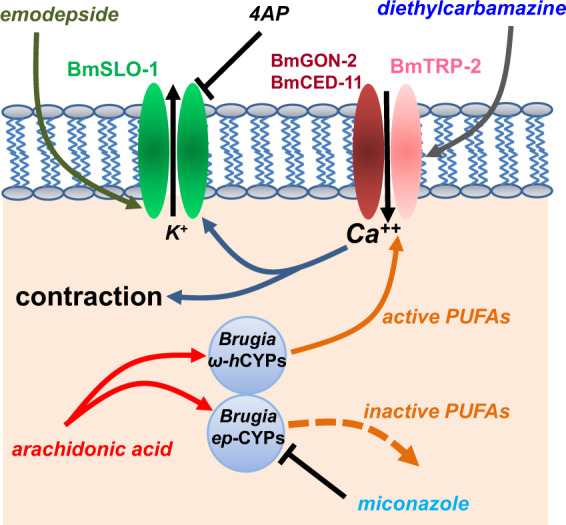
Summary diagram of the proposed actions of DEC, 4AP, emodepside, arachidonic acid, miconazole, and PUFAs (poly-unsaturated fatty acids) with effects on BmTRP channels and BmSLO-1 K channels and cytochrome oxidases (CYPs). DEC directly activates the *Brugia* TRP-2:GON-2:CED-11 channel giving rise to entry of calcium and secondary activation of calcium-activated SLO-1 K channels. Emodepside activates, and 4AP blocks the SLO-1 K channels. Arachidonic acid is metabolized to inactive PUFAs by *Brugia* epoxygenases that are inhibited by miconazole; or arachidonic acid is metabolized by ω-hydroxylases to active PUFAs that are increased in the presence of miconazole and that increase opening of TRP channels.

DEC has direct effects on *Brugia malayi* microfilariae and adults inducing spastic paralysis. Fig. 10 shows a parsimonious explanation for our observations. (1) The rapid effect of DEC inducing an inward current and secondary activation of SLO-1 K; (2) the effect of TRP antagonists; (3) the *trp-2*, *gon-2* + *ced-11* knockdown effects on currents and; (4) the effect and slow time course of arachidonic acid and miconazole inducing inward currents. We point out that the slow time course of the effect of arachidonic acid inducing an inward current does not prove that arachidonic acid, or a metabolite, activates TRP channels because arachidonic or a metabolite could induce a separate inward current and then have an inhibitory effect on the TRP current. However, Fig. 10 is a useful working model for the action of DEC.

Our observations clearly demonstrate a direct effect of therapeutically relevant DEC concentrations inhibiting motility of microfilariae and adult *B. malayi*. Until now, DEC was not understood to have direct effects on the parasites, but taken to act by stimulating host immune systems^6–8^.

Intravenous DEC^5^ produces a rapid (within minutes) decline in the level of microfilariae found circulating in the blood and a partial killing of the adults^21^. This rapid effect of DEC leads to the entrapment of the microfilariae and the adults in the liver, lymph nodes, and lungs where they undergo attack by the mononuclear phagocyte system (MPS) and lysis^22,23^. The reduction in circulating parasites is, however, transient in nature, and lasts several hours before returning to former levels. We found that the effect of DEC on microfilariae and adult females in vitro was also to produce a concentration-dependent inhibitory effect (microfilariae EC_50_ 6 µM; adult EC_50_ 3 µM) on motility with the motility returning gradually after 3–5 h (Figs. 1a, b and 2a–c). These concentrations are close to peak plasma concentrations in excess of 5 µM following treatment with DEC for lymphatic filariasis^24^ but they are below the EC_50_ for SLO-1K (14 µM) and the *EC*_*50*_ for the TRP muscle current (39 µM). This is explained by the classic phenomena of “Spare Receptors” where a full biological response (muscle paralysis) can be achieved with only a proportion of activated receptors (TRP channels here) when there is a signaling cascade following receptor activation that produces loss of muscle contractility^25^.

A number of authors attribute the therapeutic effects of DEC to be host mediated by their immune systems^8,26–28^ partly because of a lack of effects previously detected on the parasites in vitro. We think it is possible that the effects on motility have been overlooked because they are transient in nature.

Piessens & Beldekas^26^ reported effects of DEC on host eosinophils, leukocytes, and granulocytes and Cesbron et al.^27^, reported effects on blood platelets. These studies and others have been reviewed^6,7^. Our studies have not tested effects on host immune systems, so we do not exclude such effects of DEC.

However, here we have shown that DEC, at low therapeutic concentrations, produces a fast and temporary spastic paralysis of adult female *B. malayi* that includes activation of TRP channels that includes TRP-2 subunits of their muscle cells. TRP channels are tetramers composed of four subunits that are mostly homomeric but can also be heteromeric^15,29^. Most TRP channels are permeable to calcium and subjected to sophisticated auto-regulation mechanisms that self-regulate calcium entry and downstream calcium signaling^30^. We can explain the temporary spastic paralysis of *B. malayi* by an agonist action of DEC on TRP channels that is followed by auto-regulatory mechanisms leading to a subsequent closure of the TRP channel and recovery of motility. A temporary paralysis of the worms in the blood will lead to trapping of any paralyzed parasites in different capillary beds where they will undergo attack by the mononuclear phagocyte system (MPS) and immune systems that is required for the elimination of the worms. The entry of calcium also activates of *B. malayi* SLO-1 K channels (Fig. 2), that are activated by emodepside^10,31^. The anticipated effects of DEC are synergistic on the antifilarial effect of emodepside that activates SLO-1 K channels (Fig. 10). It is also possible that TRP channels, specifically TRP-2 will support the action of cholinergic anthelmintics in manner similar to the response of nicotine that is promoted by TRP-2 in *C. elegans*^32^. TRP channels are also present in the flatworm helminth, *Schistosoma mansoni*, that causes schistosomiasis, and a TRPM channel has recently been discovered to be activated by the anthelmintic, praziquantel^33^ emphasizing the recognition and importance of role of TRP channels in the action of anthelmintic drugs.

Finally, TRP channels are also present on many host cells including those involved in immune responses^34^. Entry of calcium through DEC activated host TRP channels may contribute to enhancement of an immune response to the filarial parasite, producing a DEC-induced dual parasite- and host-mediated action.

## Methods

### Parasite maintenance

*B. malayi* microfilariae and adult worms were provided by NIH/NIAID Filariasis Research Reagent Resource Center (FR3; College of Veterinary Medicine, University of Georgia, Athens, GA, USA). Worm handling protocols were approved by Institutional Bio-safety Committee (IBC) at Iowa State University. Microfilariae and adult worms were maintained in non-phenol red Roswell Park Memorial Institute (RPMI) 1640 media (Life Technologies, USA) supplemented with 10% heat-inactivated fetal bovine serum (FBS, Fisher Scientific, USA) and 1% penicillin–streptomycin (Life Technologies, USA). The worms were stored individually in 24-well culture plates containing 2 ml of supplemented RPMI −1640 media and placed in an incubator at 37 °C supplemented with 5% CO_2_.

### Drugs

Emodepside used in this study was obtained from Bayer Animal Health. Diethylcarbamazine (DEC), 4-aminopyridine, miconazole, meclofenamate, arachidonic acid, iberiotoxin, and capsaicin were obtained from Sigma Aldrich (St. Louis, MO, USA). The drugs were dissolved in distilled water or DMSO and diluted in recording solution to obtain final concentration.

### *Brugia malayi* adult and microfilariae motility screening

Microfilariae were concentrated by centrifugation (400 × *g* for 10 min) and the highly motile microfilariae were collected and quantified microscopically. Forty microfilariae were then separated into each well of the 96 well plates in 200 μl of RPMI culture media. The microfilariae were kept at 37 °C under an atmosphere of 5% CO_2_ before and after addition of DEC during experiments as per protocol described by Samje et al.^9^. The volume was restricted so that the movement of the microfilariae was restricted to two dimensions. To observe the DEC concentration effect, we applied different concentrations of DEC (100 nM, 300 nM, 1, 3, 10, 30, and 100 µM) and used distilled water as the vehicle control. The motility of the microfilariae were counted manually (0, 0.33, 0.165, 0.5, 1, 2, 24, 48, and 72 h) as the percentage of microfilariae that were motile. The microfilariae were graded as: (1) fully motile, if they showed normal movement with no coiling or, (2) immotile, if they were immotile, or contracted showing tail, head, or whole worm coiling. The percent motility of the microfilariae in the individual wells was then determined at concentrations and at different times. The percent motility at 1 h was used to determine the concentration effect plot and the EC_50_ of DEC

Movement of adult worms was analyzed in a 24-well culture plate using the Worminator video capture and motility monitoring system as described in detail^35^. Each adult worm was placed in a single well of the culture plate containing 1 mL of supplemented RPMI culture as described above. DEC concentration–response analysis, in adult worms, was done after addition of DEC (300 nM, 1, 3, 10, and 30 µM) and the motility of the worms was recorded 0, 0.5, 10, 20, 30, 40, 60, 90, 240, and 480 min post treatment. A 30 s time point was used to draw the concentration–response curve. For dsRNA experiments, the movement of worms was recorded at 0, 1, 4, 24, 48, 72, and 96 h post dsRNA treatment using the WormAssay v1.4 software. Motility of all the worms was also recorded before the application of dsRNA. The worms were treated with DEC after 96 h of RNAi to estimate the loss of drug response.

### Dissection

After dissection of the adult worms, recordings were performed at room temperature. The muscle cells and the hypodermis were exposed upon dissection by modifying the methods used for *C. elegans*^36,37^. Sections of about 5 mm were cut from the anterior region of the worm and placed in the recording chamber with bath solution with calcium (23 mM NaCl, 110 mM Na acetate, 5 mM KCl, 6 mM CaCl_2_, 4 mM MgCl_2_, 5 mM HEPES, 10 mM d-glucose, and 11 mM sucrose) or without calcium (23 mM NaCl, 110 mM Na acetate, 5 mM KCl, 10 mM MgCl_2_, 5 mM HEPES, 10 mM d-glucose, and 11 mM sucrose). The pH of both solutions was adjusted to 7.2 with NaOH and the osmolarity maintained at ~320 mOsm. The base of the chamber was a 24 × 50 mm cover slip coated with a thin layer of Sylgard^™^. The worm section was then glued along one side using Glushield^®^ cyanoacrylate glue (Glustitch Inc., Canada) thereby immobilizing it and then cut open longitudinally using a tungsten needle. The resulting “muscle flap” was glued along the cut edge and the reproductive and the gut tissue were removed using fine forceps. The dissection was viewed under DIC optics (×400) using an inverted light microscope (TE2000U, Nikon, USA).

### Whole-cell recording

Muscle flaps were incubated in 1 mg/ml collagenase (Type 1A) in bath solution for 15–120 s and washed 10 times prior to recording. The patch-clamp technique was used to record whole-cell currents from the muscle flaps as explained in ref. ^38^. Patch pipettes were pulled from capillary glass (G85150T, Warner Instruments Inc., Hamden, CT, USA), fire polished, and then filled with pipette solution with calcium (120 mM KCl, 20 mM KOH, 4 mM MgCl_2_, 5 mM TRIS, 0.25 mM CaCl_2_, 4 mM NaATP, 5 mM EGTA, and 36 mM sucrose) or without calcium (120 mM KCl, 20 mM KOH, 4 mM MgCl_2_, 5 mM TRIS, 4 mM NaATP, 5.2 mM EGTA, and 36 mM sucrose). The pH for both solutions was adjusted to 7.2 with KOH, and the osmolarity maintained at ~315–330 mOsm. Pipettes with resistances of 3–5 MΩ were used. A 1 cm region near the tip of the electrode was covered with Sylgard^™^ to reduce background noise and improve frequency responses. Giga ohm seals were formed before breaking the membrane with suction. The preparation was continuously perfused in bath solution at 2 ml/min. The current signal was amplified by an Axopatch 200B amplifier (Molecular Devices, CA, USA) filtered at 2 kHz (three-pole Bessel filter), sampled at 25 kHz, and digitized with a Digidata 1440A (Molecular Devices, CA, USA).

### RNA extraction and cDNA synthesis

Microfilariae and adult worms were snap frozen and crushed into fine powder in a 1.5 ml micro-centrifuge tube using Kimble^TM^ Kontes^TM^ Pellet Pestle^TM^ (Fisher Scientific, USA). Total RNA was extracted using TRIzol^®^ Reagent (Life Technologies, USA) according to the manufacturer’s instructions. About 1 µg of total RNA was used to synthesize cDNA using SuperScript^®^ VILO™ Master Mix (Life Technologies, USA). Samples were either used to amplify DNA using PCR or stored at −20 °C for later use.

### Single muscle cell PCR

Whole-cell patch pipettes were used to collect cytoplasm from individual somatic muscle cells for expression analysis^13,14^. cDNA was synthesized using the cytoplasm as a template using SuperScript^®^ VILO™ Master Mix (Life Technologies, USA). cDNA was later used to perform PCR reactions.

### Synthesis and delivery of dsRNA

dsRNA was synthesized as explained in refs. ^13,39^. Target and non-target T7 promoter labeled primers were amplified using the primers trp-2f2, trp-2f2t7, trp-2r2, trp-2r2t7 for *trp-2*, gon-2f1, gon-2f1t7, gon-2r1, gon-2r1t7 for *gon-2*, ced-11f1, ced-11f1t7, ced-11r1, ced-11r1t7 for *ced-11*, and osm-9f, osm9ft7, osm-9r, osm9rt7 for *osm-9*. The primers LacZF, LacZR, LacZFt7 and LacZRt7 were used to amplify the non-target LacZ. The sequences of these primers are shown in Supplementary Table S1. Amplification was performed from sequence verified cDNA templates using a Techne^TM^ PRIMEG cycler (Bibby Scientific Limited, UK) with the following cycling conditions: −95 °C × 5 min, 35 × (95 °C × 30 s, 55 °C × 30 s, 72 °C × 1 min), 72 °C × 10 min. dsRNA was synthesized using the T7 RiboMAX^TM^ Express RNAi kit (Promega, USA) according to the manufacturer’s instructions. Concentration and purity of dsRNA were assessed using a spectrophotometer. Adult *B. malayi* were soaked in 30–60 µg/ml of dsRNA for 4 days. dsRNA for the transcripts *Bma osm-9, Bma gon-2, Bma ced-11, Bma trp-2*, individually, a mix of *Bma gon-2, ced-11*, and *trp-2*, or for the control LacZ dsRNA or the control DNA/RNase free water was used for respectively. Worms were maintained in RPMI media as explained before. A part of the worm was cut for electrophysiology recordings and the rest was snap frozen in liquid nitrogen and stored at −80 °C for transcript analysis by qPCR.

### Analysis of transcript levels

cDNA from dsRNA-treated worms were amplified using the following target and reference gene (*Bma gapdh*) primers: trp-2f2, trp-2r2, gon-2f1, gon-2r1, ced-11f1, ced-11r1, osm-9f, osm-9r, SSK5F, and SSK5R (Supplementary Table S1). These genes were amplified in triplicate by quantitative real-time PCR (qPCR) using the CFX96 Touch^TM^ Real-Time PCR Detection System and SsoAdvanced^TM^ Universal SYBR® Green Supermix (Bio-Rad, USA). The cycling conditions used were 95 °C × 10 min, 40 × (95 °C × 10 s, 55 °C × 30 s). PCR efficiencies were calculated using the CFX96 Software Suite (Bio-Rad, USA). The relative quantification of target gene knockdown was estimated by the ∆∆Ct method^40^.

### Statistics and reproducibility

Whole-cell patch-clamp data were analyzed with Clampfit 11.1 (Molecular Devices, CA, USA) and GraphPad Prism 5.0 software. The peak current-responses from whole-cell recordings were used for analysis. For whole worm concentration–response relationships, motility/minute was plotted against log concentration. Drug concentrations were log_10_ transformed before analysis. The log agonist vs. response equation (variable slope) was used to generate concentration–response curves to calculate EC_50_ values. The responses were plotted as the mean ± SE. Statistical analyses were performed on groups of values by using ANOVA to determine whether the group means were dissimilar. Bonferroni post-hoc tests were used for multiple comparison tests to determine whether there were significant differences between groups. *T*-tests were used for comparing a simple control with a test study effect. Group data presented in the manuscript subjected to statistical analysis had a minimum of *n* = 5 independent samples/individuals per group, regardless of the outcome of any power analysis. Minimum sample size was 5 for all the experiments and it was also randomized for minimum of three batches of worms. Worms were randomly allocated to each treatment group by blinded operator. Motility of the worms was tested on minimum of three random batches of worms with minimum of 3 worms from each batch used. The whole-cell recordings were made on random muscle/regions of the worms. Investigators were blinded to data collection for motility recordings and analysis. For electrophysiology recordings, blinding was not possible as operator had to have the knowledge of the specimen to test the drugs and randomization was irrelevant considering our experimental design as described above.

### Reporting summary

Further information on research design is available in the Nature Research Reporting Summary linked to this article.

## Supplementary information

Supplementary Information

Supplementary Data 1

Description of Additional Supplementary Files

Reporting Summary

## Data Availability

All data generated or analyzed during this study are included in this published article and its supplementary files. Any remaining data can be obtained from the corresponding author upon reasonable request.
