# Author Correction: Diethylcarbamazine activates TRP channels including TRP-2 in filaria, *Brugia malayi*

**DOI:** 10.1038/s42003-020-01194-8

**Published:** 2020-08-12

**Authors:** Saurabh Verma, Sudhanva S. Kashyap, Alan P. Robertson, Richard J. Martin

**Affiliations:** Department of Biomedical Sciences, Iowa State University, Ames, IA 50011 USA

Correction to: *Communications Biology* 10.1038/s42003-020-01128-4, published online 28 July 2020.

In the original published version of the article, an equal contributions statement for authors Saurabh Verma and Sudhanva S. Kashyap was inadvertently omitted. The error has been corrected in the HTML and PDF versions of the article.

