# Supplementary material for: Diethylcarbamazine activates TRP channels including TRP-2 in filaria, Brugia malayi
Source: Commun Biol. 2020 Jul 28;3:398. doi: 10.1038/s42003-020-01128-4 (PMC7387335; doi:10.1038/s42003-020-01128-4)
Supplement: Supplementary file 1 — Supplementary Information [file 42003_2020_1128_MOESM1_ESM.pdf]

Supplementary Figure 1

a.

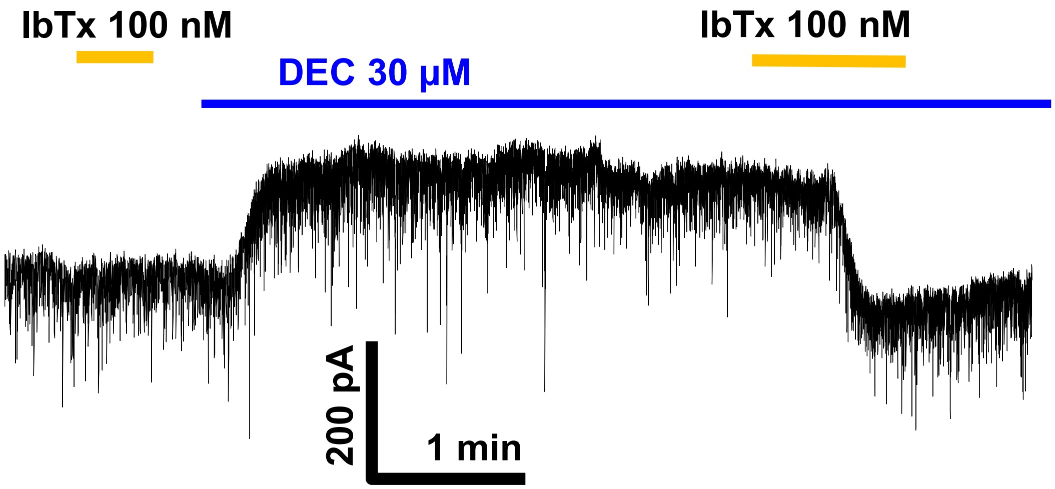

b.

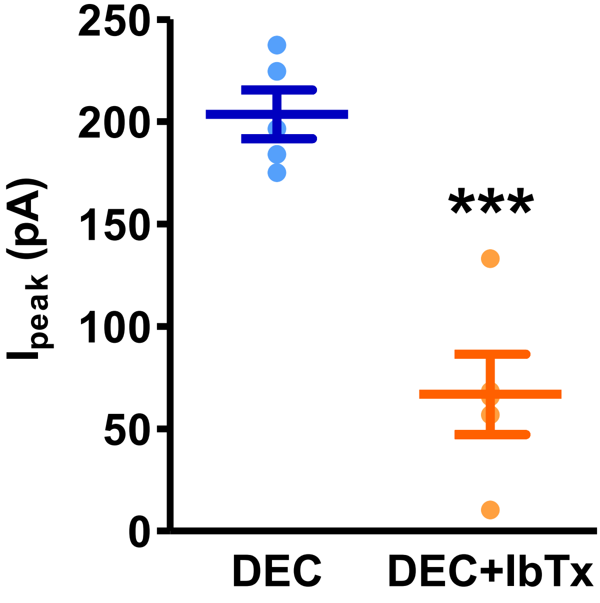

Supplementary Figure 1. BK channel blocker, iberiotoxin (IbTx), inhibits the outward potassium current evoked by DEC.

a: Representative trace of iberiotoxin having no effect on its own but inhibits the outward current produced by DEC.

b: Whisker-plot showing the reduction in the DEC outward currents (204 ± 12pA) in the presence of IbTx (67 ± 20pA, paired *t*-test, p=0.0004; 95% confidence interval was 100 to 200; n=5 from 5 worms).

Supplementary Figure 2

a.

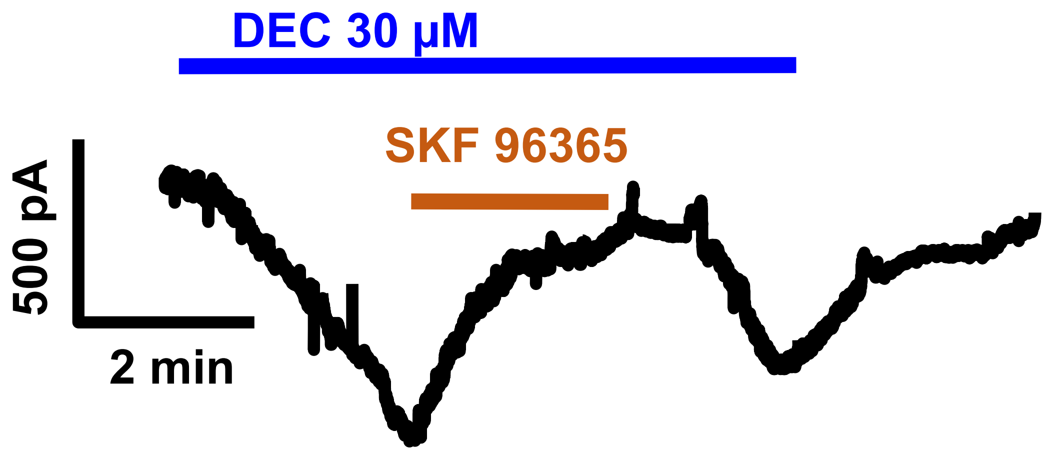

b.

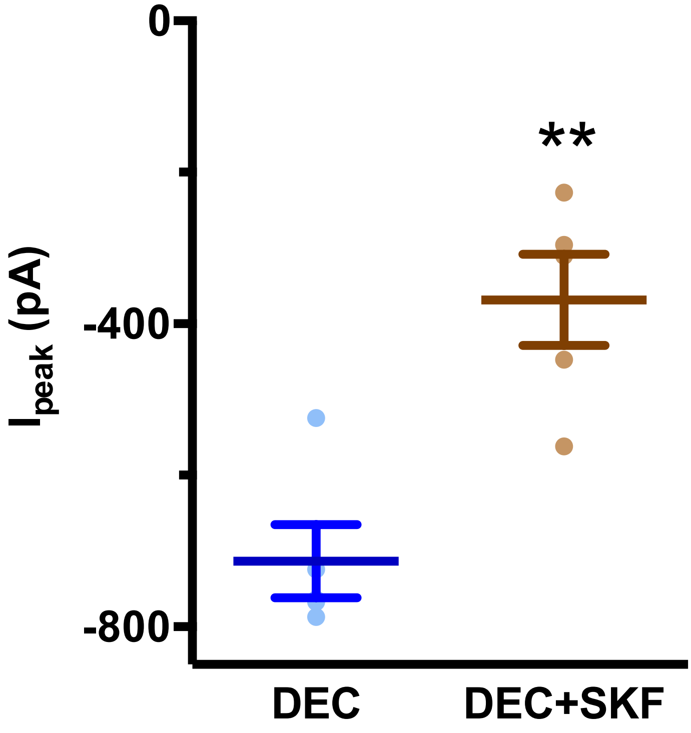

Supplementary Figure 2. SKF96365 inhibits DEC induced inward currents in the presence of 4-AP

a: Representative trace of TRP agonist SKF96365 inhibiting the DEC induced inward current in the presence of 4-AP.

b: Whisker-plot of the DEC induced inward current in the presence (-713 ± 48pA) and absence of SKF96364 (-368 ± 60pA, paired *t*-test, p=0.0058; 95% confidence interval was -500 to -200; n=5 from 5 worms).

Supplementary Figure 3

a.

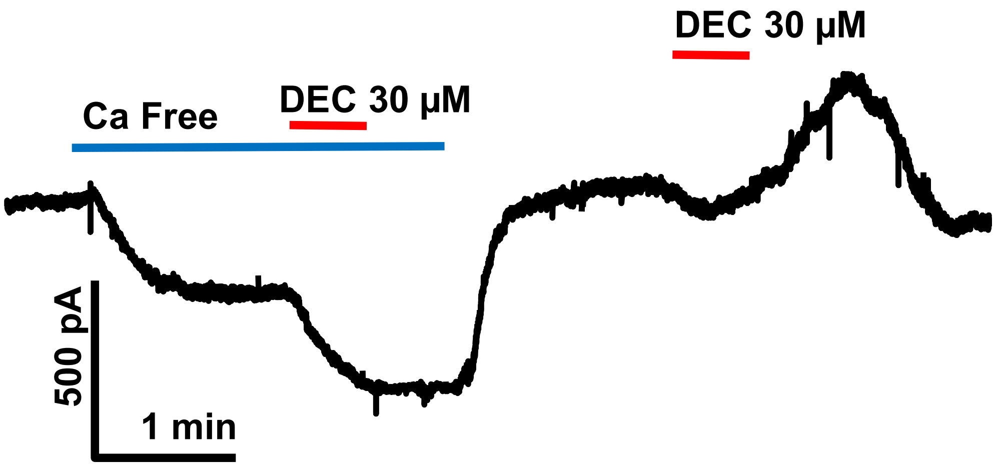

b.

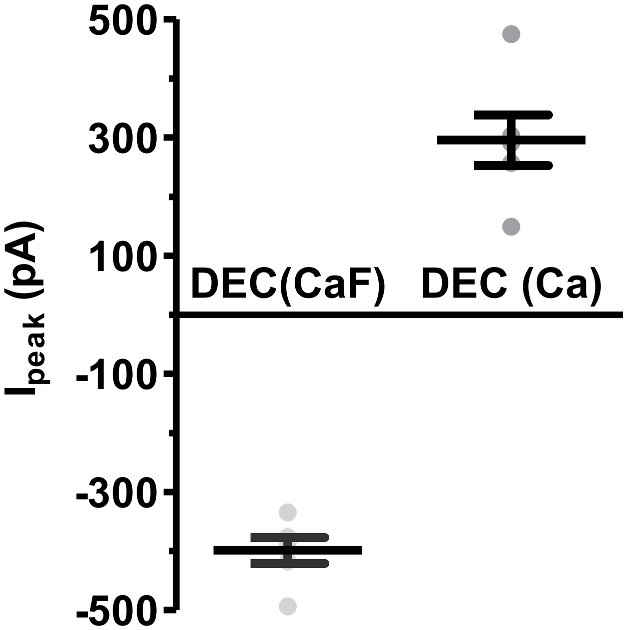

Supplementary Figure 3. DEC produces an inward current in calcium free media

a: Representative trace of DEC producing an inward current in the presence of calcium-free bath solution. This inward current changes to an outward current in the presence of calcium (Ca) and in absence of potassium channel block (n=6).

b: Box-plot showing the outward current in the presence of calcium (DEC (Ca): 295 ± 43pA) and inward current in the absence of calcium (DEC (CaF): -398 ± 22 pA; n=6 from 6 worms).

Supplementary Figure 4

a.

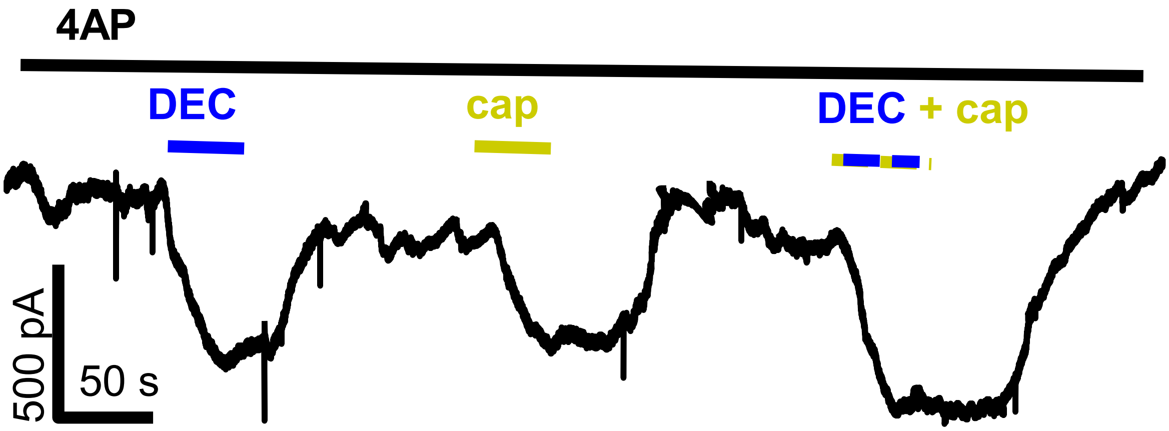

b.

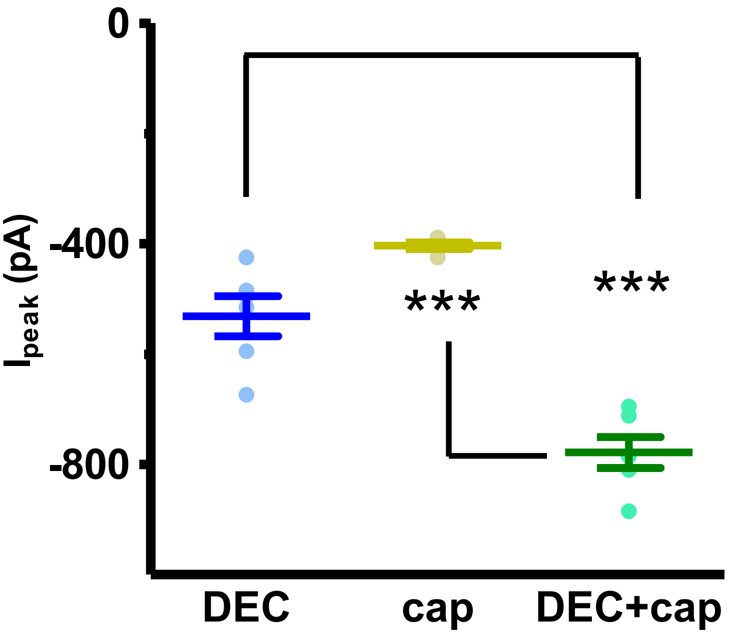

Supplementary Figure 4. Capsaicin (cap) induces inward currents similar to DEC in the presence of 5 mM 4AP.

a: Representative inward current following the application of 30μM DEC, 30μM capsaicin and 30μM (DEC + capsaicin).

b: Whisker-plot demonstrating inward currents induced by DEC (-531 ± 36pA), capsaicin (-403.5 ±6pA) and DEC+ capsaicin (-778 ± 28.2pA) respectively (One way ANOVA; $p<0.0001$; 95% confidence interval for DEC vs DEC+ capsaicin was 100 to 300 and capsaicin vs DEC+ capsaicin was 300 to 500 ; n=6 from 6 worms).

Supplementary Figure 5

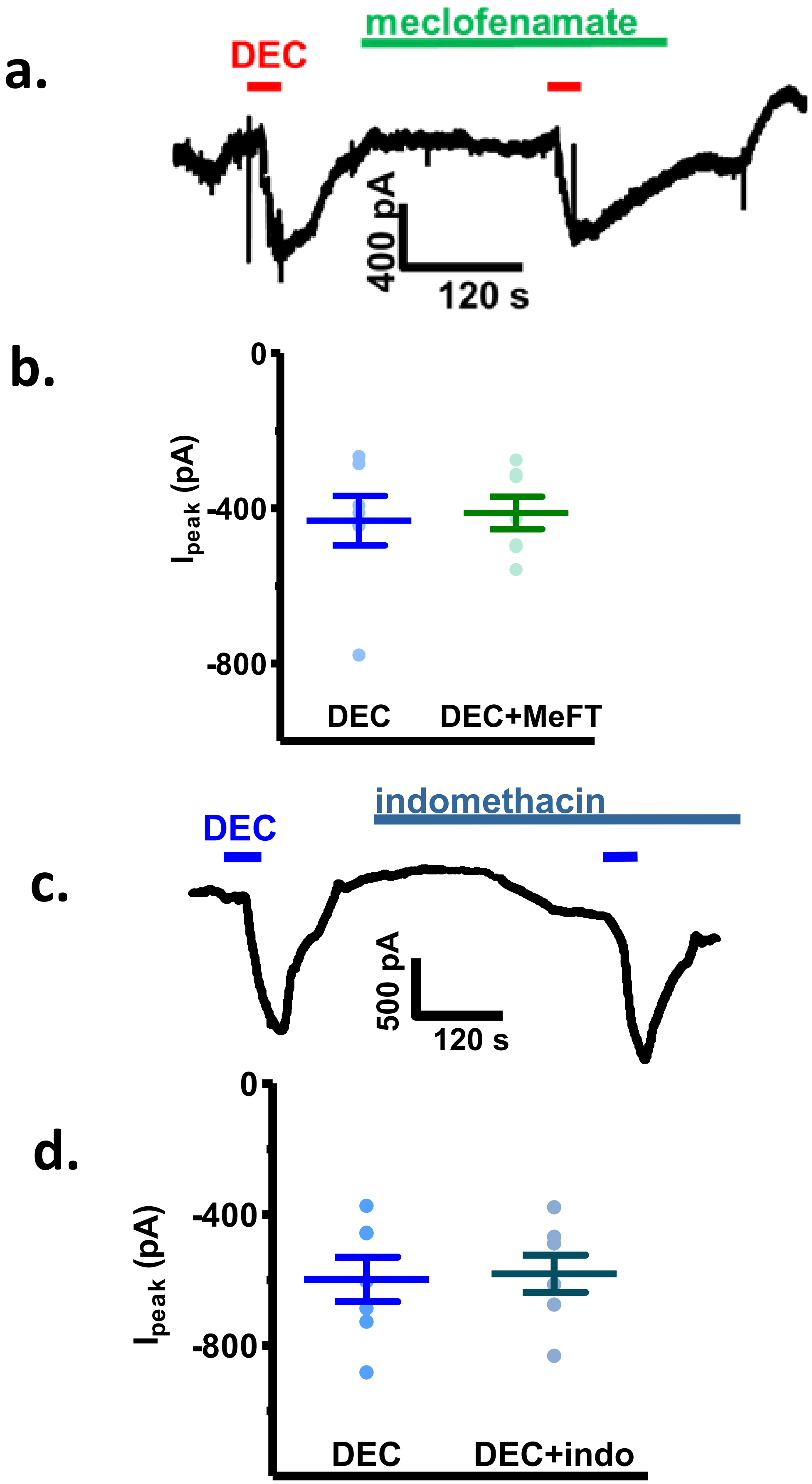

Supplementary Figure 5. Effects of meclofenamate (MeFt) and indomethacin (indo) on the DEC response DEC in the presence of 5 mM 4AP.

a: Representative inward current following the application of 30μM DEC, 10μM meclofenamate and 30μM DEC in the presence of 10μM meclofenamate. Note the absence of an effect of meclofenamate.

b: Whisker-plot showing the inward currents induced by 30μM DEC (DEC: -446 ± 77pA) and by 30μM DEC in the presence of 10 μM meclofenamate (DEC+MeFT: -431 ± 55pA) showing no significant change (95% confidence interval for DEC vs DEC+MeFT was -100 to 70, paired *t*-test; p=0.671; n=7 from 6 worms).

c: Representative inward currents following the application of 30μM DEC, followed by the addition of 30μM DEC in the presence of 30μM indomethacin.

Table S1: List of primers used in this study

Primer Name	Description	Sequence 5' - 3'
**gon-2f1**	Bma gon-2 5’	GGGTGGAACAAACATTCAAA
**gon-2r1**	Bma gon-2 3’	TTCGGCTTCTTCATCACTTC
**ced-11f1**	Bma ced-11 5’	ATTGATGCACCACAAAGTGA
**ced-11r1**	Bma ced-11 3’	TGCCACACTGTAAATGTGCT
**trp-2f2**	Bma trp-2 5’	AAGAAGTACGTGGACCACCA
**trp-2r2**	Bma trp-2 3’	TCGAAGTGCAACGGTACATA
**ocr-1f**	Bma ocr-1 5’	TCGGAATAAAGAGCGAAAT
**ocr-1r**	Bma ocr-1 3’	AGTTTCCACGTCACTTCTCG
**osm-9f**	Bma osm-9 5’	CCGCAAATGATTCGTTTTAC
**osm-9r**	Bma osm-9 3’	ACACCACGTTTGTTCAACCT
**cup-5f**	Bma cup-5 5’	CTTCGAGCTCATCTCCTGAA
**cup-5r**	Bma cup-5 3’	TGCAAAGAGTGCCACAGATA
**trpa-2f**	Bma trpa-2 5’	CAGAGACATGCGACTGCTTA
**trpa-2r**	Bma trpa-2 3’	TCGGTGATATTTTGCTTGGT
**GAPDHf**	Bma GAPDH 5'	GACGCTTCAAGGGAAGTGTTTCTG
**GAPDHr**	Bma GAPDH 3'	GTTTTGGCCAGCACCACGAC
**LacZf**	LacZ dsRNA 5'	CGTAATCATGGTCATAGCTGTTTC
**LacZr**	LacZR dsRNA 3'	CTTTTGCTGGCCTTTTGCTC
**LacZft7**	LacZ dsRNA with t7 promoter 5'	TAATACGACTCACTATAGGGCGTAATCATGGTCATAGCTGTTTC
**LacZrt7**	LacZ dsRNA with t7 promoter 3'	TAATACGACTCACTATAGGGCTTTTGCTGGCCTTTTGCTC
