# Supplementary material for: Diethylcarbamazine activates TRP channels including TRP-2 in filaria, Brugia malayi
Source: Commun Biol. 2020 Jul 28;3:398. doi: 10.1038/s42003-020-01128-4 (PMC7387335; doi:10.1038/s42003-020-01128-4)
Supplement: Supplementary file 3 — Description of Additional Supplementary Files [file 42003_2020_1128_MOESM3_ESM.pdf]

Description of Additional Supplementary Files

File Name: **Supplementary Data 1**
Description: Raw data Excel files
